# Supplementary figures and images for: Cardiopulmonary bypass in a rat model may shorten the lifespan of stored red blood cells by activating caspase-3
Source: PLoS One. 2023 Sep 20;18(9):e0290295. doi: 10.1371/journal.pone.0290295 (PMC10511131; doi:10.1371/journal.pone.0290295)

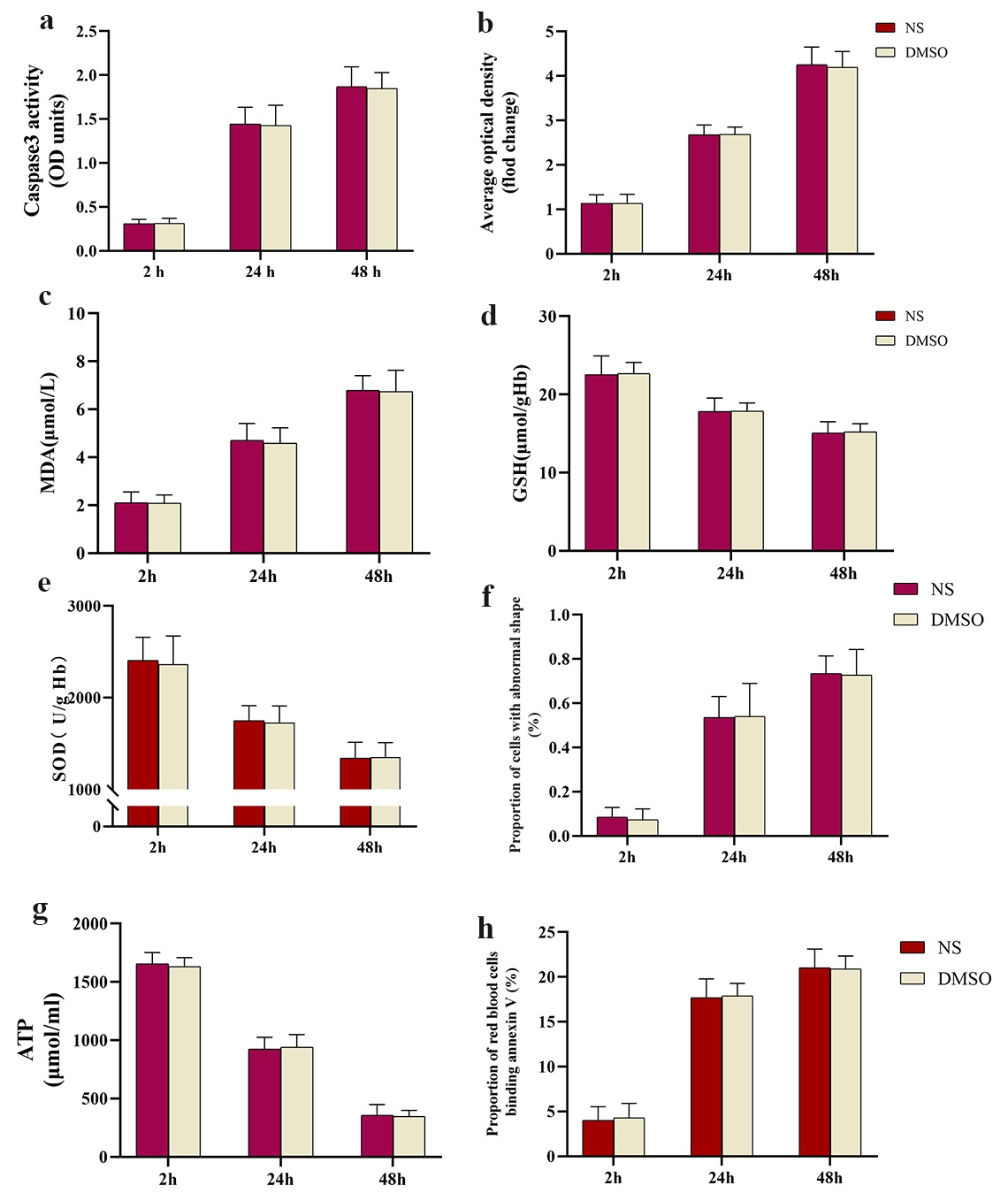

Supplement: S1 Fig — At the indicated time points, the suspension was assayed for (a) caspase-3, (b) reactive oxygen species (ROS), (c) malonic dialdehyde (MDA), (d) L-glutathione (GSH), (e) superoxide dismutase (SOD), (f) abnormal morphology, (g) ATP, and (h) ability to bind annexin V. Data are mean ± SD (n = 10 animals per condition). OD, optical density. (TIF) [file pone.0290295.s001.tif]
