# Supplementary material for: Cardiopulmonary bypass in a rat model may shorten the lifespan of stored red blood cells by activating caspase-3
Source: PLoS One. 2023 Sep 20;18(9):e0290295. doi: 10.1371/journal.pone.0290295 (PMC10511131; doi:10.1371/journal.pone.0290295)
Supplement: Attachment — Submitted filename: Response to Reviewers.docx [file pone.0290295.s003.docx]

11 June 2023

Dear Dr Pidcoke:

We would like to thank you for the time spent on our manuscript “Cardiopulmonary bypass in a rat model may shorten the lifespan of stored red blood cells by activating caspase-3” (PONE-D-23-00813). Below we provide point-by-point responses to referees’ comments, and we have marked the major revisions in the manuscript in blue.

We hope the manuscript can now be judged suitable for publication in the *PLOS ONE*. We would be happy to respond to any further questions or comments that you or the reviewers may have.

Sincerely,

Daming Gou

Department of Anesthesiology

KweiChow Moutai Hospital

Renhuai, Guizhou, China

Tel: +86-13985219675

E-mail: [gdmzy@yeah.net](mailto:gdmzy@yeah.net)

**RESPONSES TO COMMENTS FROM EDITOR:**

1. Please ensure that your manuscript meets PLOS ONE's style requirements, including those for file naming. The PLOS ONE style templates can be found at [https://journals.plos.org/plosone/s/file?id=wjVg/PLOSOne_formatting_sample_main_body.pdf and](https://journals.plos.org/plosone/s/file?id=wjVg/PLOSOne_formatting_sample_main_body.pdf%20and) <https://journals.plos.org/plosone/s/file?id=ba62/PLOSOne_formatting_sample_title_authors_affiliations.pdf>

**Response:** We have revised our manuscript according to the PLOS ONE's style requirements.

2. Thank you for stating the following in the Acknowledgments Section of your manuscript:

"This work was supported by funds from the National Natural Science Foundation of China (81860083). The authors are grateful to the participants for their interest and cooperation. "

We note that you have provided additional information within the Acknowledgements Section that is not currently declared in your Funding Statement. Please note that funding information should not appear in the Acknowledgments section or other areas of your manuscript. We will only publish funding information present in the Funding Statement section of the online submission form.

Please remove any funding-related text from the manuscript and let us know how you would like to update your Funding Statement. Currently, your Funding Statement reads as follows:

"This work was supported by funds from the National Natural Science Foundation of China (81860083). The funders had no role in study design, data collection and analysis, decision to publish, or preparation of the manuscript."

Please include your amended statements within your cover letter; we will change the online submission form on your behalf.

**Response:** We have updated our Funding Statement.

3. "Please amend the manuscript submission data (via Edit Submission) to include author "Lianlian Li , Hangya Linghu, Lei Zheng , Daming Gou"

**Response:** We have revised the manuscript submission data.

4. We note that you have included the phrase “data not shown” in your manuscript. Unfortunately, this does not meet our data sharing requirements. PLOS does not permit references to inaccessible data. We require that authors provide all relevant data within the paper, Supporting Information files, or in an acceptable, public repository. Please add a citation to support this phrase or upload the data that corresponds with these findings to a stable repository (such as Figshare or Dryad) and provide and URLs, DOIs, or accession numbers that may be used to access these data. Or, if the data are not a core part of the research being presented in your study, we ask that you remove the phrase that refers to these data

**Response:** We have deleted the phrase “data not shown” in our manuscript.

5. Please include your full ethics statement in the ‘Methods’ section of your manuscript file. In your statement, please include the full name of the IRB or ethics committee who approved or waived your study, as well as whether or not you obtained informed written or verbal consent. If consent was waived for your study, please include this information in your statement as well.

**Response:** We have added the full ethics statement in the ‘Methods’ section of our manuscript file (lines 69-71).

6. In your Data Availability statement, you have not specified where the minimal data set underlying the results described in your manuscript can be found. PLOS defines a study's minimal data set as the underlying data used to reach the conclusions drawn in the manuscript and any additional data required to replicate the reported study findings in their entirety. All PLOS journals require that the minimal data set be made fully available. For more information about our data policy, please see http://journals.plos.org/plosone/s/data-availability.

Upon re-submitting your revised manuscript, please upload your study’s minimal underlying data set as either Supporting Information files or to a stable, public repository and include the relevant URLs, DOIs, or accession numbers within your revised cover letter. For a list of acceptable repositories, please see http://journals.plos.org/plosone/s/data-availability#loc-recommended-repositories. Any potentially identifying patient information must be fully anonymized.

Important: If there are ethical or legal restrictions to sharing your data publicly, please explain these restrictions in detail. Please see our guidelines for more information on what we consider unacceptable restrictions to publicly sharing data: http://journals.plos.org/plosone/s/data-availability#loc-unacceptable-data-access-restrictions. Note that it is not acceptable for the authors to be the sole named individuals responsible for ensuring data access.

We will update your Data Availability statement to reflect the information you provide in your cover letter.

**Response:** We have added supporting information S1 table as minimal data set to support our results described in manuscript.

7. Please review your reference list to ensure that it is complete and correct. If you have cited papers that have been retracted, please include the rationale for doing so in the manuscript text, or remove these references and replace them with relevant current references. Any changes to the reference list should be mentioned in the rebuttal letter that accompanies your revised manuscript. If you need to cite a retracted article, indicate the article’s retracted status in the References list and also include a citation and full reference for the retraction notice.

**Response:** We have reviewed and corrected the relevant literatures.

**RESPONSES TO COMMENTS FROM REVIEWER** **1:**

1. These experiments utilize stored red cells which are likely to have developed storage lesions and be more fragile than fresh cells. Would freshly collected red cells produce the same results in these experiments? What storage time of human red cells does 10 days of rat red cell storage represent? This could be mentioned in the discussion.

**Response:** We now mention that the 5-day storage period of the rat red blood cells in our experiments is comparable to 10-day storage of human red blood cells, which is comparable to the average duration of storage before use in the clinic (line 301-303).

2. Free radicals have a very brief life span. How is it possible that plasma from by-pass animals could retain the free radicals? If the free radicals remain in the plasma it should be possible to include anti-oxidant molecule(s) that would block the free radical effect on red cells when they are incubated with the by-pass plasma. Such an experiment would strengthen the free radical argument. Alternatively, there could be other explanations such as complement activation and/or free hemoglobin release. These too could be measured in the by-pass plasma. Similarly performing the experiments with fresh cells which may be less prone to lysis would reduce the possibility that free hemoglobin is involved in the responses.

**Response:** We thank the reviewer for these insightful comments, which we suggest as important hypotheses to examine in future work (lines 335-337 and lines 348-351).

3. Phagocytosis of red cells FITC labels only the outside of red cells. Physical stress/damage from the by-pass pumps likely leads to release red cell membrane fragments or microvesicles while the red cell remains in circulation. In several places it is stated that macrophages and neutrophils can internalize damaged RBCs because there is increased fluorescence associated with these cells. An alternate explanation could be that these cells are internalizing membrane fragments or microvesicles labelled with FITCs or that the fragments and microvesicles are attached to the outside of the cells. Fluorescent microscopy should be able to determine which process is going on.

**Response:** In our hands, FITC labels both the membrane and cytoplasm of red cells (Figure 1, *below*). We agree with the reviewer that the macrophages and neutrophils may have acquired FITC labeling by phagocytosing entire red blood cells or membrane fragments or microvesicles of red blood cells. We now mention this possibility on line 200-203.

**Figure 1.** Representative fluorescence micrographs from our experiments, in which red blood cells in suspension were labeled with FITC. The area boxed in red on the *left* is shown at higher magnification at *right*.

**RESPONSES TO COMMENTS FROM REVIEWER 2:**

1. The statement that “the function of transfused cells appears to decline over time” (L19) or “after surgery” (L46) and other similar statements present throughout the manuscript are not literally true. We know that the recovery of stored RBCs varies as a function of numerous donor- and recipient-related factors and that a percentage of transfused cells are removed from the circulation the first 24h post transfusion. However, the “survivals” circulate for a normal period in the recipient. Moreover, 24h recovery is actually the most measured property of transfused RBCs per se. In contrast, other physiological measures of transfused RBCs (deformability, morphology, fragility etc) are only indirectly assessed in vivo by measurements performed in the mixed RBC population of the recipient which consists of donor and recipient cells. We must take into consideration that these different cell populations may mutually change as a result of the transfusion event, that also includes residual donor plasma and transmittance of biological response modifiers, such as extracellular vesicles. In vitro models, like the one used by the authors, can reveal transfusion effects exclusively on donor RBCs but only those related to body temperature and recipient plasma. Despite useful and informative, these models are not equal to a “real” transfusion event.

**Response:** We appreciate the limitations that the reviewer points out in our modeling approach, which we have highlighted in the limitations section of the Discussion (lines 352-353). Nevertheless, we believe that our transfusion model is valuable for elucidating molecular and cellular details of the events affecting the lifespan of transfused red blood cells, and we were careful to validate our findings through *in vivo* experiments.

2. Figure 4 results (L242): In the basic experiment reported in the text (L239-243) stored RBC pretreated with caspase-3 inhibitor and then incubated with bypass or sham bypass plasma, while Fig 4 shows another comparison, namely that between inhibitor vs. DMSO treated RBCs with bypass plasma. First of all, DMSO seems to significantly affect the caspase activity (Fig. 2f vs. Fig. 4a).

Moreover, ROS staining shown in the representative micrographs of Fig. 4b is inconsistent with the ROS bar graphs shown in Fig. 4c. And even for the comparison DMSO vs. inhibitor, one would expect lower ROS and MDA levels in the inhibitor-treated cells (vs. the DMSO-treated ones) in the context of significantly lower caspase activity (Fig. 4a). However, this is not the case as shown in Fig. 4c-f. Having these in mind, the authors cannot support that the inhibitor reversed the effects of bypass plasma on the ROS, MDA, GSH and SOD levels as reported in L242-243.

On the opposite, the results shown Fig. 5 are consistent with lower shape abnormalities and PS exposure on cells that exhibit lower caspase activation.

**Response:** We thank the reviewer for careful consideration of our data, which led us to detect a mistake in the original Figure 4. We have now provided the correct image in the revised manuscript and revised line 244-246 to read “To examine whether bypass-induced activation of caspase-3 could explain the observed shorter lifespan of stored red blood cells, we pretreated them with the caspase-3 inhibitor Z-DEVD-FMK before mixing them with plasma from rats in bypass”.

In response to the reviewer’s comment, we incubated rat blood cells (after 5-day storage) in culture medium containing 0.9% normal saline or DMSO for 2 h, then mixed them for 48 h with bypass plasma. We found that levels of caspase-3, ROS, MDA, GSH and SOD were similar between both types of cultures during 48 h (Figure 2a-e, *below*). The same was observed for cellular morphology, ATP levels and extent of PS exposure (Figure 2f-h, *below*). We conclude that DMSO exerted negligible effects on red blood cells in our *in vitro* experiments.

We agree with the reviewer that Z-DEVD-FMK minimally affected oxidative stress in red blood cells, as shown in the revised Figure 4b-f. We have revised the text accordingly (lines 254-255).

**Figure 2.** Stored red blood cells were incubated with DMSO or normal saline (NS), then incubated in bypass plasma. At the indicated time points, the suspension was assayed for (a) caspase-3, (b) reactive oxygen species (ROS), (c) malonic dialdehyde (MDA), (d) l-glutathione (GSH), (e) superoxide dismutase (SOD), (f) abnormal morphology, (g) ATP, and (h) ability to bind annexin V. Data are mean ± SD (n = 10 animals per condition). OD, optical density.

3. According to the results L271-273 the inhibition of caspase-3 cannot reverse all the negative effects of bypass as reported in the discussion L296-297. Please rephrase.

**Response:** We have revised the sentence to “Our results further suggest that inhibiting caspase-3 can, at least partly, reverse these negative effects of bypass” (line 299-300).

4. Title: L341-342: Beyond doubt, there are additional pathways involved in RBC destruction (including the band-3/IgG/C3b and CD47-related surface removal signaling) and the authors should report them along with the respective citations.

**Response:** We now mention these pathways and corresponding references (line 337-342).

**Minor comments**

1. Introduction is too short.

**Response:** We have extended the introduction with relevant background.

2. A study limitations paragraph is missing.

**Response:** We have added discussion of study limitations (lines 343-355).

3. In my opinion, red arrow in Fig. 3 indicates an echinocyte and not an acanthocyte.

**Response:** We thank the reviewer for this careful insight. We have changed the annotation to “echinocyte”.

4. There is a mismatch in the a-f labeling between the Figure 4 plates/graphs and the Legend.

**Response:** As noted above, we have revised the Figure 4 and corresponding legend.

5. The two sentences in L271-274 need rephrasing.

**Response:** We have revised these sentences (lines 276-281).

6. “It has been shown that caspase-3 is present in red blood cells…” Another work to support this statement in stored RBCs is that of Kriebardis et al. (TRANSFUSION 2007; 47:1212-1220).

**Response:** We have added this as ref 23 (lines 454-457).
